# Epigenetic mechanisms involved in differential *MDR1 *mRNA expression between gastric and colon cancer cell lines and rationales for clinical chemotherapy

**DOI:** 10.1186/1471-230X-8-33

**Published:** 2008-08-01

**Authors:** Tae-Bum Lee, Jung-Hee Park, Young-Don Min, Kyung-Jong Kim, Cheol-Hee Choi

**Affiliations:** 1 Research Center for Resistant Cells, Chosun University, Gwangju 501-759, Korea; 2 Department of Pharmacology, Chosun University Medical School, Gwangju 501-759, Korea; 3 Department of Surgery, Chosun University Medical School, Gwangju 501-759, Korea

## Abstract

**Background:**

The membrane transporters such as P-glycoprotein (Pgp), the *MDR1 *gene product, are one of causes of treatment failure in cancer patients. In this study, the epigenetic mechanisms involved in differential *MDR1 *mRNA expression were compared between 10 gastric and 9 colon cancer cell lines.

**Methods:**

The *MDR1 *mRNA levels were determined using PCR and real-time PCR assays after reverse transcription. Cytotoxicity was performed using the MTT assay. Methylation status was explored by quantification PCR-based methylation and bisulfite DNA sequencing analyses.

**Results:**

The *MDR1 *mRNA levels obtained by 35 cycles of RT-PCR in gastric cancer cells were just comparable to those obtained by 22 cycles of RT-PCR in colon cancer cells. Real-time RT-PCR analysis revealed that *MDR1 *mRNA was not detected in the 10 gastric cancer cell lines but variable *MDR1 *mRNA levels in 7 of 9 colon cancer cell lines except the SNU-C5 and HT-29 cells. MTT assay showed that Pgp inhibitors such as cyclosporine A, verapamil and PSC833 sensitized Colo320HSR (colon, highest *MDR1 *expression) but not SNU-668 (gastric, highest) and SNU-C5 (gastric, no expression) to paclitaxel. Quantification PCR-based methylation analysis revealed that 90% of gastric cancer cells, and 33% of colon cancer cells were methylated, which were completely matched with the results obtained by bisulfite DNA sequencing analysis. 5-aza-2'-deoxcytidine (5AC, a DNA methyltransferase inhibitor) increased the *MDR1 *mRNA levels in 60% of gastric cells, and in 11% of colon cancer cells. Trichostatin A (TSA, histone deacetylase inhibitor) increased the *MDR1 *mRNA levels in 70% of gastric cancer cells and 55% of colon cancer cells. The combined treatment of 5AC with TSA increased the *MDR1 *mRNA levels additively in 20% of gastric cancer cells, but synergistically in 40% of gastric and 11% of colon cancer cells.

**Conclusion:**

These results indicate that the *MDR1 *mRNA levels in gastric cancer cells are significantly lower than those in colon cancer cells, which is at least in part due to different epigenetic regulations such as DNA methylation and/or histone deacetylation. These results can provide a better understanding of the efficacy of combined chemotherapy as well as their oral bioavailability.

## Background

Gastric and colorectal cancers are a cause of morbidity and mortality in the world today. If a curative surgical resection is impossible, these cancers respond very poorly to chemotherapy and resulting in a poor prognosis. In gastric cancer patients, 5-fluorouracil (5-FU) based combination chemotherapy have been attempted in order to improve the treatment outcomes [1]. With colorectal cancer, 5-FU has been the most widely used drug for more than 40 years. However, other agents such as irinotecan or oxaliplatin have been used to improve the antitumor efficacy in combination with 5-FU [2]. 5-FU interferes with DNA synthesis by blocking the production of pyrimidine nucleotide dTMP from dUMP during *de novo *DNA synthesis through the inhibition of thymidylate synthase as well as through the incorporation of fluoro-nucleotides into the DNA and RNA [3].

P-glycoprotein (Pgp) encoded by the multidrug resistance 1 (*MDR1*) gene is a representative membrane efflux pump of ATP-binding cassette (ABC) transporters [4-6]. Pgp functions as energy-dependent efflux pumps of a variety of structurally diverse chemotherapeutic agents such as doxorubicin, vincristine, vinblastine, paclitaxel, colhicine, actinomycin D and mitomycin C [7], which can decrease the intracellular level of drug accumulation. As a result, overexpression of these proteins confers MDR to cancer cells by evading the cytotoxic effects of drugs. In the human intestine, Pgp is strongly expressed on the apical surface of the superficial columnar epithelial cells of the ileum and colon, and its expression level decreases gradually proximally into the jejunum, duodenum and stomach [8]. Regulation of the transcriptional activity of the *MDR1 *gene is dependent on several trans-acting proteins that bind the consensus cis-elements [9]. The accessibility of the promoter elements to their binding factors is regulated at the level of chromatin assembly. The levels of both DNA methylation and histone deacetylation regulate *MDR1 *gene expression [10-12]. So far, the transcriptional regulation of *MDR1 *gene expression through epigenetic mechanisms has been reported in expression in colon cancer cells [13-16] but none in gastric cancers cells. Furthermore, the relationships between the transcriptional expression of *MDR1 *gene expression and epigenetic mechanisms in gastric and colon cancer cells have not been compared. Therefore, it is unclear why chemotherapy regimens have been differently used to treat gastric and colorectal cancers and why *MDR1 *mRNA is expressed differentially in gastric and colorectal cancer cells. Therefore, this study examined whether or not the degree of methylation at the promoter site of the *MDR1 *gene is closely associated with *MDR1 *gene expression in both cancer cells.

## Methods

### Cell culture

The 10 human gastric cancer cell lines (SNU-1, -5, -16, -216, -484, -601, -620, -638, -668 and -719) and 9 colon cancer cell lines (SNU-C1, -C4, -C5, Colo320HSR, LoVo, DLD-1, HT-29, HCT-8 and HCT-116) were obtained from the Cancer Research Center at Seoul National University (South Korea). All the cells were cultured at 37°C in a 5% CO_2 _atmosphere using RPMI 1640 medium (GibcoBRL, Gland Island, NY, USA) with 10% heat inactivated fetal bovine serum (Sigma, ST. Louis, MO, USA). The cells were maintained either as a suspension or a monolayer culture, and subcultured until they reached confluence.

### Reverse transcription-polymerase chain reaction (RT-PCR) assay

The total RNA was extracted using MagExtractor^® ^for the MFX-2100 (Toyobo, Osaka, Japan) auto-nucleic acid purification system, according to the manufacturer's instructions. The *MDR1 *and *β-actin *mRNA transcripts were detected using the RT-PCR assay. *MDR1 *expression was detected with the 5' and 3' primers corresponding to the nucleotides 907–930 (5'-CTGGTTTGATGTGCACGATGTTGG-3') and 1179–1201 (5'-TGCCAAG-ACCTCTTCAGCTACTG-3'), respectively, of the published cDNA sequence [17], yielding a 296-bp PCR product. β*-actin *mRNA expression was used as a control for the amount of RNA, and was detected with the 5' and 3' primers corresponding to nucleotides 1912–1932 (5'-GACTATGACTTAGTTGCGTTA-3') and 2392–2412 (5'-GTTGAACTCTCTACATACTTCCG-3'), respectively, of the published cDNA sequence [18], yielding a 501-bp PCR product. The RNA from each sample was reverse transcribed using 200 units of Moloney murine leukemia virus reverse transcriptase (Gibco-Bethesta Research Laboratory, Grand Island, NY, USA) and 0.18 μg/ml oligo (dT_20_) primer for 1 hr at 37°C. The resulting cDNA of the gastric cancer cells (2-fold diluted cDNA in the colon cancer cells) were amplified with 1.25 units of Taq polymerase (PE Applied Biosystems, Foster City, CA, USA), 1 mM MgCl_2 _and 10 pmole of each primer in a thermal cycler (GeneAmp 2400, PE Applied Biosystems, Boston, MA, USA) for 22 cycles with the colon cancer cells but 35 cycles with the gastric cancer cells for *MDR1 *and 17 cycles for β*-actin *of the sequential denaturation (94°C for 30 s), annealing (65°C for *MDR1*, 53°C for β*-actin*), and extension (72°C for 30 s). After the final cycle, all the PCR products were subjected to a final extension of 5 min at 72°C. For quantitation, 3 μCi of [α-^32^P] dCTP were added to each reaction mixture. After PCR, the PCR products were combined and then electrophoresed on a 7.5% nondenaturing polyacrylamide gel. The bands were scanned with a densitometer (Pdi, Huntington Station, NY, USA). The amount of each mRNA transcript was normalized with that of each β*-actin *mRNA.

### Protein extraction and Western blot analysis

Total cell lysates were prepared by lysing harvested cells in extraction buffer (1% NP-40, 0.5% sodium deoxycholate, 0.1% SDS in phosphate-buffered saline) supplemented with 2 mM phenylmethylsulfonyl fluoride (Sigma) and 10 μg/ml leupeptin (Sigma). DNA was sheared by sonication and Western blotting analysis was performed using a slight modification of the method first described by Towbin et al. [19]. Proteins were transferred onto a nitrocellulose membrane by electroblotting using a current of 60 V overnight. The membrane was incubated in blocking solution (5% skim milk) for 1 hr at room temperature, washed, and then incubated with primary goat polyclonal antibody (1:1000, Santa Cruz, Biotechnology, CA, USA) for Pgp. The membrane was washed and incubated with horseradish peroxidase-conjugated secondary antibody (diluted 1:1000) against each IgG for hosts of primary antibodies for 1 hr. The membrane was then stained using the detection reagent of the ECL detection kit (Amersham, Piscataway, NJ, USA).

### Real-time PCR

Extraction of mRNA was performed according to the RNeasy proctocol (Qiagen, Hilden, Germany). One microgram of total RNA was reversely transcribed into cDNA in a volume of 20 μl with 200 units of Moloney murine leukemia virus reverse transcriptase (Gibco-Bethesta Research Laboratory, Grand Island, NY, USA) and 0.18 μg/ml oligo (dT_20_) primers (Promega, Madison, USA) according to the manufacture's manual. Real-time PCR was performed with the Light Cycler 2.0 Instrument (Roche, Mannheim, Germany) using the Fast Start DNA Master SYBR Green I Kit (Roche). For verification of the correct amplification product, PCRs were analyzed on a 2% agarose gel stained with ethidium bromide. The sequences of the primers are as follows: for *β-actin*, 5'-GACTATGACTTAGTTGCGTTA-3' and 5'-GTTGAACTCTCTACATACTTCCG-3'; for *MDR1*, 5'-CTGGTTTGATGTGCACGATGTTGG-3' and 5'-TGCCAAGACCTCTTCAGCTACTG-3'. Each reaction (20 μl) contained 4 μl cDNA (10-fold dilution), 4 mM MgCl_2_, 10 pmole of each primer and 2 μl of Fast Start DNA Master SYGR Green I Mix containing buffer, dNTPs, SYBR Green dye and Tag polymerase. The amplification procedure of target genes was as follows: pre-denaturing at 95°C for 10 min, 40 cycles of denaturing at 95°C for 15 sec, annealing for *MDR1 *at 67°C (*β-actin *at 55°C) for 5 sec, and extension at 72°C for 7 sec (*β-actin *for 21 sec). Melting curve analysis was performed to confirm production of a single product. Negative controls without template were produced for each run. Gene expression values (relative mRNA levels) are expressed as ratios (difference between the Ct values = The point on the curve in which the amount of fluorescence begins to increase rapidly, usually a few standard deviations above the baseline, is termed the threshold cycle (Ct value).) between the gene of interest (*MDR1 *mRNA) and an internal reference gene (*β-actin *mRNA) that provide a normalization factor for the amount of RNA isolated from a specimen. Analysis of data was performed using Light Cycler software version 4.0 (Roche).

### Cytotoxicity test using MTT assay

The *in vitro *cytotoxicity of the drugs was measured using an MTT assay, as described elsewhere [20]. The cells were seeded at a 2 × 10^4^cells/ml and incubated overnight to allow for attachment and stabilization. The cells were incubated at 37°C for 3 days, and MTT [3-(4,5-dimethylthiazole-2-yl)-2,5-diphenyl tetrazolium bromide, Sigma] solution was then added to each well containing the cells. After shaking for 1 min, the plate was incubated for 5 hr. Formazan crystals of the suspension culture were dissolved in 150 μl of dimethylsulfoxide (DMSO) after removing the supernatant. The optical density of the wells was measured with a microplate reader (μQuant, Bio-tek Instruments Inc., Winooski, VT, USA) at 540 nm.

### Quantification PCR-based methylation analysis

Five micrograms of the genomic DNA was digested with 50 U of *Msp *I or *Hpa *II (Fermentas MBI, Vilnius, Lithuania) at 37°C for 16 hours, added to a 1/15 volume of 0.6 M Tris (pH 7.5) and 1.5 M NaCl, and digested with 50 U of *Pst *I (New England Biolabs, MA, USA) at 37°C for 8 hours. The methylation status of the *MDR1 *5'CpG promoter region was examined by analyzing 100 ng restriction-digested DNA by PCR in 25 μL reactions containing 1.25 units of Taq DNA polymerase and 10 pmole of each primer. The quantification PCR-based methylation analysis was carried out according to the method reported previously [11]. The PCR primers used were 5'-TCTAGAGAGGTGCAACGGAAG-3' and 5'-TCAGCCTCACCACAGATGAC-3' for the MS1 methylation-sensitive primers (121 bp), 5'-TGAAGTCCTCTGGCAAGTCC-3' and 5'-ATTCTCCCTCCCGGTTCC-3' for the MS2 methylation-sensitive primers (206 bp), 5'-ATTTCACGTCTTGGTGGCC-3' and 5'-TCCAGTGCCACTACGGTTT-G-3'for the MC positive control primers (240 bp), and 5'-GGCGAAGGAGGT-TGTCTATTC-3' and 5'-AACGTTCTAGGAGAGTCGGG-3' for MN negative control primers (240 bp) derived from the triosephosphate isomerase gene promoter region. Amplification was performed in a DNA thermal cycler for 35 cycles for the MN, MC, MS1 and MS2 primers involving in sequence denaturation (95°C for 30 s), annealing (60°C for 30 s), and extension (72°C for 30 s). After the final cycle, all the PCR products were subjected to a final extension for 5 min at 72°C. The PCR products were separated by electrophoresis on 7% PAGE gels. The gels were then stained with ethidium bromide, and photographed by using a Kodak Image Station 4000 MM (Eastman Kodak, Rochester, NY, USA).

### Bisulfite DNA sequencing analysis

One μg of genomic DNA was chemically modified by sodium bisulfite using the EZ DNA Methylation kit (Zymo Research, Orange, CA, USA) to convert unmethylated cytosines to uracils while leaving methylated cytosines unaltered. The bisulfite-modified DNA was used for PCR amplification. Extended MS1 primer contains 10 CpG sites, and 2 SP-1 sites which are mandatory for the functional *MDR1 *promoter to be activated [21]. The primer sequences for amplification of bisulfite-treated strands (223 bp) were as follows: S: 5'-GGAAGTTAGAATATTTTTTTTGGAAAT-3'; AS: 5'-ACCTCTACTTCTTTAAACTTAAAAAAACC-3'. Amplification was performed in the same PCR conditions except 48°C annealing temperature and 45 PCR cycles. After the final cycle, all the PCR products were subjected to a final extension at 72°C for 5 min. Sequence of PCR products was analyzed using an automated sequencer (Applied Biosystems, Foster City, CA, USA).

### Statistical Analysis

The results are presented as a mean ± SE and the data was analyzed using the Student's *t-test*. P values < 0.05 were considered significant.

## Results

### Comparison of expression profiles of MDR1 mRNA in gastric and colon cancer cells

**Figure 1:**
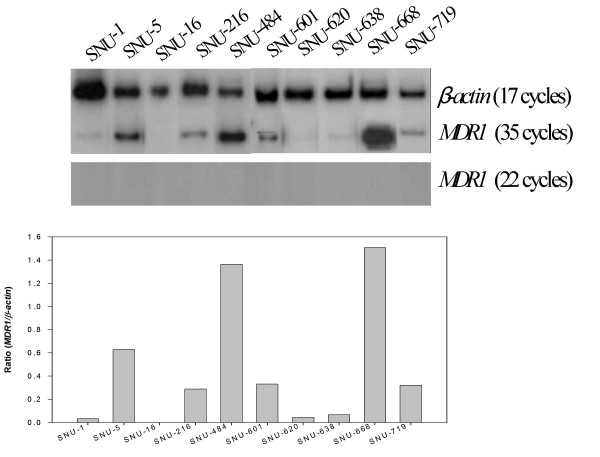
***MDR1 *mRNA expression in gastric cancer cell lines**. The level of *MDR1 *mRNA expression was determined by RT-PCR, and normalized by that of mRNA *β-actin*, which was used as a control for RNA. The cDNA reverse-transcribed from the mRNA was amplified separately with each primer pair for *MDR1 *and *β-actin *genes. Aliquots of each PCR reaction mixture were separated on 7% polyacrylamide gel. The gel was dried and exposed on X-ray film overnight.

**Figure 2:**
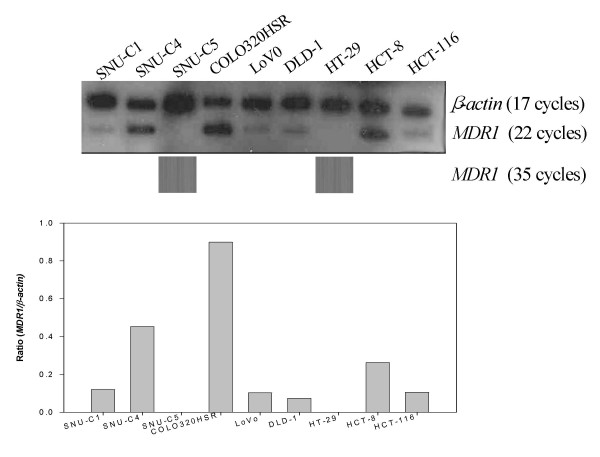
***MDR1 *mRNA expression in the colon cancer cell lines**. The same methodology reported in Figure 1 was used.

*MDR1 *mRNA expression was analyzed using the RT-PCR assay with the expression level being normalized with the *β-actin *mRNA levels obtained after 17 cycles of PCR. The *MDR1 *mRNA was not detected after 22 cycles of PCR in the 10 gastric cancer cell lines but could be detected at variable levels after 35 cycles of PCR with the exception of SNU-16, suggesting a significantly low level of *MDR1 *mRNA expression in the gastric cancer cells tested (Figure 1). As shown in Figure 1, the rank order according to the *MDR1-β-actin *ratio in the gastric cancer cell lines is as follows: SNU-668 (1.51) > SNU-484 (1.37) > SNU-5 (0.63) > SNU-601 (0.33) > SNU-719 (0.32) > SNU-216 (0.29) > SNU-638 (0.07) > SNU-1 (0.04) > SNU-16 (0). Of the 9 colon cancer cell lines, variable *MDR1 *mRNA levels could be detected in 7 colon cancer cell lines after 22 cycles of PCR but not in the SNU-C5 and HT-29 cells. The *MDR1 *mRNAs of the two latter cells could be not detected even after 35 cycles of PCR. These results suggest a relatively high level of *MDR1 *mRNA expression in spite of some exceptions in the colon cancer cells. As shown in Figure 2, the rank order according to the *MDR1*/*β-actin *ratio in the colon cancer cell lines is as follows: Colo320HSR (0.90) > SNU-C4 (0.45) > HCT-8 (0.26) > SNU-C1 (0.12) > HCT-116 (0.11) > LoVo (0.10) > DLD-1 (0.07) > SNU-C5 (0) = HT-29 (0).

**Figure 3:**
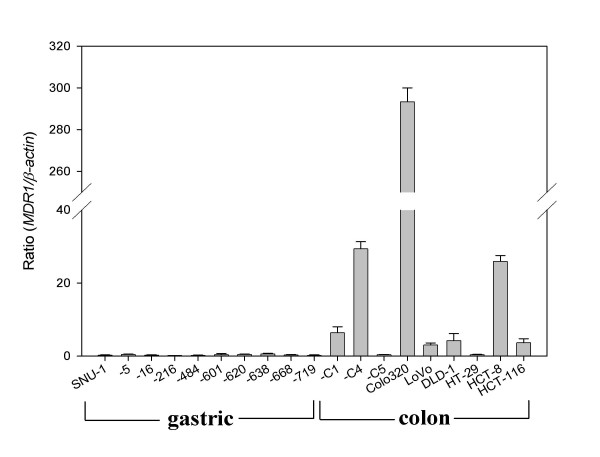
***MDR1 *mRNA expression in gastric and colon cancer cell lines**. The level of *MDR1 *mRNA expression was determined by real-time RT-PCR, and normalized by that of mRNA *β-actin*, which was used as a control for RNA. The cDNA reverse-transcribed from the mRNA was amplified separately with each primer pair for *MDR1 *and *β-actin *genes.

We performed again the real-time RT-PCR assay for the quantitative validation of *MDR1 *mRNA levels obtained from RT-PCR assay. The *MDR1 *mRNA was not detected in the 10 gastric cancer cell lines. However, of the 9 colon cancer cell lines, variable *MDR1 *mRNA levels could be detected in 7 colon cancer cell lines except the SNU-C5 and HT-29 cells as the RT-PCR data (Figure 3).

Taken together, the *MDR1 *mRNA levels in the gastric cancer cell lines were significantly lower than those in the colon cancer cell lines.

### Chemosensitizing effects of Pgp inhibitors in gastric and colon cancer cells

**Figure 4:**
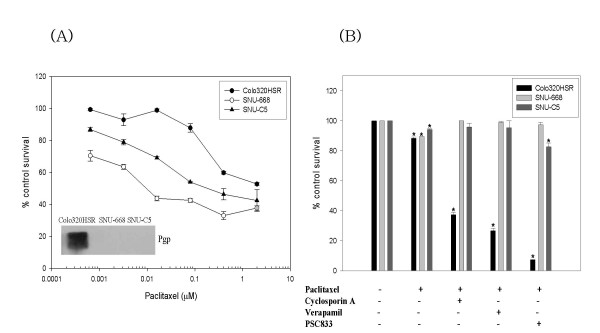
**Comparison of Pgp expression and function in gastric and colon cancer cell lines**. (A) Comparative sensitivity of Colo320HSR (colon, highest), SNU-668 (gastric, highest) and SNU-C5 (colon, no expression) to paclitaxel; (B) Effects of Pgp inhibitors on the sensitivity of Colo320HSR, SNU-668 and SNU-C5 to paclitaxel (IC10 concentration; 50 μM, 0.3 nM and 0.5 nM, respectively). Sensitivity to paclitaxel was determined using MTT assay in the presence or absence of the Pgp inhibitors (cyclosporin A, verapamil and PSC833 of 0.8 μM each). *, P <0.05 versus the control.

Although the protein levels were not examined in this study, functional studies were performed using the Pgp inhibitors in the gastric and colon cancer cell lines expressing the highest level of *MDR1 *mRNA expression. As shown in Figure 4A, the Colo320HSR cells (colon, mutant p53, highest expression of *MDR1 *mRNA) were 14-fold and > 200 times resistant to paclitaxel than the SNU-C5 and SNU-668 cells (gastric, mutant p53, highest expression of *MDR1 *mRNA) as compared on the basis of the IC_50 _values, respectively, representing a significant difference in the Pgp levels. In addition, the resistance of the Colo320HSR cells to paclitaxel was reversed by the Pgp inhibitors including cyclosporine A, verapamil, and PSC833 (Figure 4B). However, this reversal was not observed in the SNU-C5 (colon, no *MDR1 *mRNA) cells as well as SNU-668. This suggests that Pgp expressed in colon cancer cells but not gastric cancer cells works functionally and can be inhibited by the Pgp inhibitors.

### Comparison of methylation status of MDR1 in gastric and colon cancer cells

**Figure 5:**
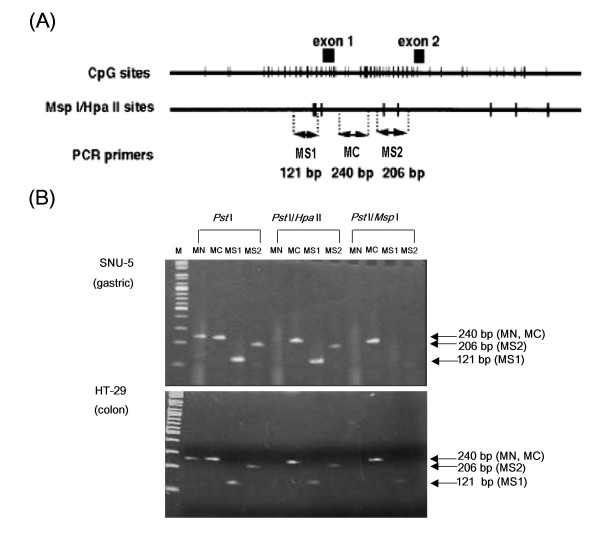
**The quantification PCR-based methylation analysis of gastric and colon cancer cell lines**. (A) CpG sites and *Hpa II*/*Msp I *sites in the human *MDR1 *promoter region. Top: The CpG sites are represented by the short vertical bars. The positions of exons 1 and 2 are indicated as closed boxes. The position corresponding to these fragments are indicated. Middle: *Hpa II*/*Msp I *recognition sites are represented by short vertical bars. Bottom, PCR primers used in methylation analysis. (B) Representative methylation status of the *MDR1 *promoter region by quantification PCR-based methylation analysis in SNU-5 (gastric) and HT-29 (colon). 1: MN, Never-methylated *Hpa II*/*Msp I *site at the triosephosphate isomerase gene promoter region (negative control) (240 bp); 2: MC, the positive control primer pair (240 bp); 3: MS1, *Hpa II*/*Msp I *site 1 (121 bp); 4: MS2, *Hpa II*/*Msp I *site 2 (206 bp).

The methylation status was determined by quantification PCR-based methylation analysis for a CpG-rich domain to be approximately 1 Kb containing exon 1 and intron 1 among the *MDR1 *promoter. To determine the degree of methylation of the *MDR1 *gene promoter region, two primers (MS1 and MS2) containing the *Msp *I/*Hpa *II sites were designed from exon 1 and intron 1, respectively. The primer pair MC was used as a positive control to determine the quality of the source genomic DNA. In contrast, the MN that crosses the *Msp *I/*Hpa *II site at the triosephosphate isomerase gene promoter region and is never methylated was used as the negative control. Figure 5B shows typical quantification PCR-based methylation analysis images of the SNU-5 (gastric) and HT-29 (colon) cells. The quantification PCR-based methylation analysis revealed that any PCR products for the MS1 and MS2 were not produced from *Pst*1-digested genomic DNA treated with *Msp *I (methylation-insensitive enzyme). On the other hand, PCR products for both MS1 and MS2 in the SNU-5 cells but a PCR product for the MS1 alone in the HT-29 cells were obtained after *Hpa *II (methylation-sensitive enzyme) treatment, indicating methylation of CpG at the MS1 and MS2 sites in the SNU-5 cells and only at the MS2 site in the HT-29 cells. As summarized in Table 1, methylation was detected at the MS1 and MS2 sites of the 9 gastric cancer cell lines with the exception of SNU-484 but 2 (SNU-C5 and HCT-116) of the 9 colon cancer cell lines. On the other hand, the HT-29 cells were methylated only at the MS2 site. The SNU-C5, HT-29 (colon) and SNU-16 (gastric) cells not expressing *MDR1 *mRNA were methylated. Bisulfite DNA sequencing analysis was performed to confirm the methylation. As show in Table 1, methylation degree (%) of 10 CpG sites on the expended MS1 site is completely matched with results obtained by quantification PCR-based methylation analysis.

**Table 1:** Methylation status and the effects of 5AC and/or TSA on *MDR1 *mRNA expression and in various gastric and colon cancer cell lines

				DNA methylation assay						
								
Tissue	Cell line	5AC		Restriction Enzyme		Sodium Bisulfite	TSA		5AC +TSA	
						
		Fold	Effect	Site		Degree (%)^1^	Fold	Effect	Fold	Effect
Gastric	SNU-1	32.6	O	MS1	MS2	100	20.7	O	+23.6	D
	SNU-5	5.8	O	MS1	MS2	100	1.03	X	6.8	A
	SNU-16	n.d.	X	MS1	MS2	60	8.4	O	28.9	S
	SNU-216	-1.0	X	MS1	MS2	50	8.4	O	8.2	N
	SNU-484	-1.3	X	-	-	0	-5.1	X	-0.17	-
	SNU-601	3.2	O	MS1	MS2	100	3.3	O	13.7	S
	SNU-620	67.6	O	MS1	MS2	100	*	X	*	*
	SNU-638	68.9	O	MS1	MS2	100	5.7	O	72.9	A
	SNU-668	-1.0	X	MS1	MS2	80	1.8	O	4.4	S
	SNU-719	5.8	O	MS1	MS2	100	4.2	O	12.4	S

Colon	SNU-C1	-1.96	X	n.d.	n.d.	0	1.7	O	+1.0	D
	SNU-C4	1.0	X	n.d.	n.d.	0	-1	X	-1.6	N
	SNU-C5	n.d.	X	MS1	MS2	100	n.d.	X	8	S
	Colo320HSR	1.4	X	n.d.	n.d.	0	1.6	O	+1.3	D
	LoVo	-1.9	X	n.d.	n.d.	0	1.1	X	-2	N
	DLD-1	1.1	X	n.d.	n.d.	0	3.5	O	+1.1	D
	HT-29	*	X	n.d.	MS2	0	∞	O	*	*
	HCT-8	1.0	X	n.d.	n.d.	0	1.0	X	1.1	N
	HCT-116	1.7	O	MS1	MS2	100	1.6	O	1.6	N

^1^Sequence was analyzed using PCR products containing the extended MS1 site that has been shown to play an important role in *MDR1 *mRNA expression. n.d., not detected; *, highly cytotoxic; ∞, an increase as compared with no *MDR1 mRNA *expression; D, decreased; A, additive; S, synergistic; N, not changed

### Effects of 5-aza-2'-deoxcytidine (5AC) and/or trichostatin A (TSA) on the expression of MDR1 mRNA in gastric and colon cancer cell lines

**Figure 6:**
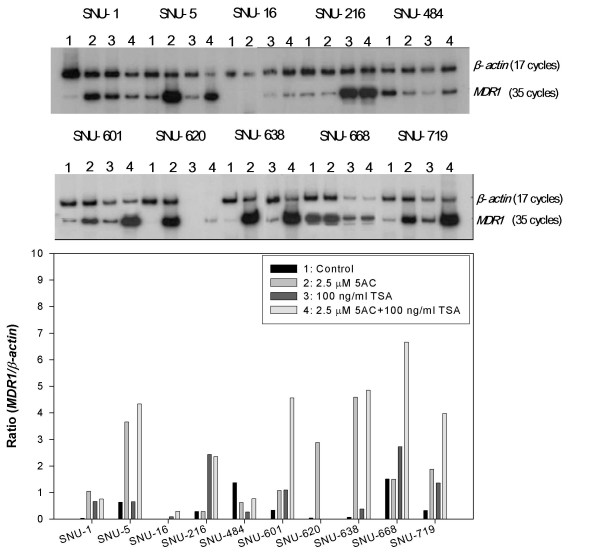
**Activation of *MDR1 *mRNA expression by 5AC and/or TSA in gastric cancer cells**. The expression level is reported as the ratio of *MDR1*/β-actin. The total RNA was isolated after treatment with 2.5 μM 5AC for 96 hr and/or 100 ng/ml TSA for 48 hr. RT-PCR was performed using the same methodology reported in Figure 1.

**Figure 7:**
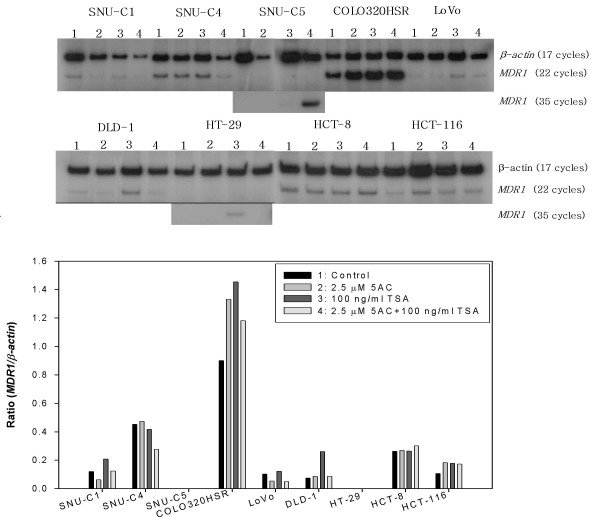
**Activation of *MDR1 *mRNA expression by 5AC and/or TSA in various colon cancer cell lines**. RT-PCR assay after treating the cells with 5AC and/or TSA using the same method described in Figure 6. The *MDR1/β-actin *ratio obtained through 35-cycle PCR after TSA in combination with 5AC, and alone in SNU-C5 and HT-29 expressing no *MDR1 *mRNA, respectively, was omitted in the histogram.

The DNA methyltransferase inhibitor 5AC and the histone deacetylase (HDAC) inhibitor TSA have been well known to relieve epigenetic gene repression [22]. This study examined the effect of 5AC and/or TSA on *MDR1 *mRNA expression in the gastric and colon cancer lines. In 10 gastric and 9 colon cancer cells, *MDR1 *mRNA expression was determined by RT-PCR after treating them with 2.5 μM 5AC for 96 hr and/or 100 ng/ml TSA for 48 hr. An increase was defined in cases showing more than a 1.5-fold increase. As shown in Table 1, the 5AC treatment increased the *MDR1 *mRNA levels in the SNU-1, -5, -601, -620, -638 and -719 gastric cancer cell lines, and that in the HCT-116 colon cancer cell line (Figures 6 and 7). However, 5AC did not induce *MDR1 *mRNA expression even in the SNU-16 and SNU-C5 and HT-29 cells whose *MDR1 *gene was methylated. The TSA treatment increased the *MDR1 *mRNA levels in the SNU-1, -16, -216, -601, -638, -668 and -719 gastric cancer cell lines and the SNU-C1, Colo320HSR, DLD-1, H29 and HCT-116 colon cancer cell lines (Figure 6 and 7). 5AC showed high cytotoxicity alone or in combination with TSA, particularly in the HT-29 cells. Also, TSA showed highly cytotoxic activity alone or in combination with 5AC, particularly in the SNU-620 cells. This study also examined the effects of the combined treatment of 5AC with TSA, which increased the *MDR1 *mRNA levels additively in the SNU-5 and -638 cells but synergistically in the SNU-16, -601, -668, -719 and SNU-C5 cells (Table 1).

These results suggest that *MDR1 *mRNA expression is differentially regulated in gastric and colon cancer cells with respect to the silencing of *MDR1 *expression through epigenetic mechanisms such as DNA methylation and/or histone deacetylation.

## Discussion

In this study, we found that the *MDR1 *mRNA levels in the gastric cancer cell lines were significantly lower than those in the colon cancer cell lines, although there were some variations. These results are consistent with a report showing that Pgp is strongly expressed on the ileum and colon, at a level that gradually decreases proximally into the jejunum, duodenum and stomach [8]. Since the stomach and colon play major roles in digestion and absorption, respectively, it is not surprising that transporters such as Pgp were differentially expressed in the two normal tissues. Our finding that the differential expression of *MDR1 *mRNA in cancer cell lines derived from the stomach and colon is also consistent with published reports [23-26]. Immunopathological studies revealed that Pgp expression on human tumors was most commonly detected in colon, renal, and adrenal carcinomas but rarely in lung and gastric carcinomas and certain germ cell tumors [27].

The three-way connection between DNA methylation, chromatin structure and gene expression was recently reviewed [28-30]. An important consequence of CpG methylation is the local silencing of gene expression, which can be mediated by the direct interference of methylation with the binding of various transcription factors. The major component of silencing of gene expression appears to be the binding of methyl-CpG-binding protein 2 (MeCp2), which has an affinity for methyl-CpG [31,32]. DNA demethylation by 5AC causes the release of the MeCp2 from the promoter, which activates transcriptional gene expression [10]. It is known that MeCp2 is also enriched on the *MDR1 *promoter and is related to its silencing [33]. 5AC alters the methylation pattern of the *MDR*1 promoter in Pgp-negative cells to resemble that of Pgp-positive cells and activates the promoter such that *MDR1 *mRNA is detectable [34].

In this study, the methylation status was also analyzed in order to determine if the *MDR1 *silencing is due to hypermethylation of the promoter region. Quantification PCR-based methylation analysis showed methylation in 9 (90%) out of 10 gastric cancer cell lines but only 3 (33%) out of 9 colon cancer cells, which were completely matched with the results obtained by bisulfite DNA sequencing assay. The latter frequency is relatively high compared with a different study showing *MDR1 *methylation in 24% of 275 colorectal cancers [16]. As showed in Table 1, complete but not partial methylation in the extended MS1 site was responsible for increased *MDR1 *mRNA expression by the treatment with 5AC. In addition, MS1 site derived from exon 1 of *MDR1 *promoter has shown to be more important with respect to gene expression than MS2 site from intron 1 of *MDR1 *promoter.

The histone-modifying enzymes such as histone acetyltransferase (HAT) and HDAC enzymes also modulate transcription of *MDR1 *[15]. Therefore, we have investigated how epigenetic mechanisms, such as DNA methylation and histone deacetylation, are involved in the differential expression of *MDR1 *mRNA between gastric and colon cancer cells using 5AC and/or TSA. The following summarizes the results obtained after the 5AC and/or TSA treatment. Effects of 5AC and/or TSA are defined as positive when > 1.5-fold is increased after treatment.

1) In gastric cancer cells, 5AC and TSA induced *MDR1 *mRNA expression at a frequency of 6/10 (60%) and 7/10 (70%), respectively. On the other hand, in colon cancer cells, 5AC and TSA induced *MDR1 *mRNA expression at a frequency of 1/9 (11%) and 5/9 (55%), respectively. This suggest that DNA methylation is at least partly responsible for the low level of *MDR1 *mRNA expression in gastric cancer cells but is rarely involved in colon cancer cells whereas HDAC may play important roles in *MDR1 *mRNA expression in both cells.

2) 5AC alone had no effect but combined with TSA synergistically increased the *MDR1 *mRNA expression level in 20% (SNU-16 and -668) of gastric cancer cells but only the SNU-C5 colon cancer cells. This suggests that the expression of a methylated *MDR1 *gene insensitive to 5AC alone increased with the assistance of TSA. This result is consistent with a previous report that silencing conferred by MeCp2 and methylated DNA can be also relieved by inhibition of HDAC, facilitating the remodelling of chromatin and transcriptional activation [35]. Although TSA alone cannot activate hypermethylted *MDR1 *[10], it can lead to upregulation of non-methylated or sparely methylated promoters [36]. Thus, epigenetic modifications of DNA and histone have been shown to be responsible for *MDR1 *gene silencing. However, it is still unclear which one is first, DNA methylation or histone modifications [37]. Moreover, combined effect of 5AC with TSA was less than that of 5AC or TSA alone in gastric (SNU-1) and colon (SNU-C1, Colo320HSR and DLD-1) cancer cells, indicating a more complex relation between the methylated DNA and HDAC.

3) TSA but not 5AC induced *MDR1 *mRNA expression in 30% (SNU 16, -216 and – 668) of gastric cancer cells and 40% (SNU-C1, COLO32HSR, DLD-1 and HT-29) of colon cancer cells. These findings suggest that HDAC is dominant over DNA methylation in cancer cells whose *MDR1 *genes are not methylated. However, synergistic effects of TSA when combined with 5AC showing no effect in gastric cancer cells (SNU-16 and -668) with unmethylated *MDR1*gene are not fully understood. The possibility of involvement of histone methylation in silencing of *MDR1 *expression remains to be determined.

4) The combined treatment of 5AC with TSA increased *MDR1 *mRNA expression either additively 20% (SNU-5 and -638) or synergistically 40% (SNU-16, -601, -668 and -719) in the gastric cancer cells but only synergistically in the SNU-C5 colon cancer cells. The synergistic effect of 5AC and TSA in gastric cancer cells can be explained on the basis of a report showing that the methylated gene binds MeCP2, which in turn recruits HDAC resulting in the suppression of transcription [32,35].

5) Neither 5AC nor TSA induced *MDR1 *mRNA expression even in gastric (SNU-484) and colon (SNU-C4, -C5 and Lovo) cancer cells even though combination of 5AC and TSA increased *MDR1 *mRNA expression in the SNU-C5 cells. This suggests the involvement of other factors in *MDR1 *mRNA expression or inappropriate concentrations and incubation period of each inhibitor.

One of the aims of this study was to explain why the different 5-FU-based anticancer therapies have been used in gastric and colorectal cancers using differential *MDR1 *expression. It has been well known that high levels of thymidylate synthase activity are responsible for the resistance to 5-FU [38]. However, antimetabolites such as 5-FU are not substrates for the ATP-dependent efflux transporters such as Pgp expressed on the apical (brush-border) membrane of intestinal epithelial cells [39-41]. Therefore, the *in vitro *and *in vivo *anticancer efficacy of 5-FU can be explained not only by the increase in its intracellular accumulation in cancer cells but also by the enhancement of its bioavailability when administered orally. In gastric cancer, even anthracyclines (doxorubicin or epirubicin) and mitomycin C, which are good Pgp substrates, have been used to treat gastric cancer cells, which are characterized by zero or low levels of Pgp, which would make them sensitive to these anticancer drugs. In colon cancer, a number of novel anticancer drugs including oxaliplatin and irinotecan have been used in various combinations [3]. Platinum compounds such as oxaliplatin, which are not substrates for Pgp and breast cancer resistance protein (BCRP), have been used as effective agents against colorectal cancer. Even though irinotecan is a BCRP substrate, it has been shown to be effective in colon cancers with a significantly lower BCRP expression level than that of the normal colon [42]. Nevertheless, it is essential that substrate drugs (topotecan, irinotecan, anthracyclines, mitomycin C and trimetrexate) for Pgp and/or BCRPS used clinically in colon cancer be administered in conjunction with chemosensitizers (VX-710 [43], GF120918 [44,45], and XR-9576 [46,47]), which can reverse both Pgp and BCRP.

## Conclusion

The *MDR1 *mRNA levels in the gastric cancer cell lines were significantly lower than those in the colon cancer cell lines, which is at least in part due to differential epigenetic regulations such as DNA methylation and/or HDAC. Therefore, *MDR1/*Pgp plays more important roles in the transporting function in colon cancer cells than in gastric cancer cells. These results can provide a better understanding of the efficacy of combined chemotherapy as well as their oral bioavailability.

## Competing interests

The authors declare that they have no competing interests.

## Authors' contributions

T-BL: Study execution, data analysis and manuscript preparation. J-HP: Study execution, data analysis and manuscript preparation. Y-DM: Concept, study design and clinical considerations for gastric cancer. K-JK: Concept, study design and clinical considerations for colon cancer. C-HC: Idea, study design and manuscript preparation. All authors have read and approved the final manuscript.

## Pre-publication history

The pre-publication history for this paper can be accessed here:


